# Effect of Rehabilitation in a Dog with Delayed Recovery following TPLO: A Case Report

**DOI:** 10.3390/ani13172778

**Published:** 2023-08-31

**Authors:** Shin-Ho Lee, Jae-Hyeon Cho, Chung-Hui Kim, Dongbin Lee

**Affiliations:** 1 Department of Companion Animal Care, Kyungnam College of Information & Technology, Busan 47011, Republic of Korea; hovet519@naver.com; 2 Institute of Animal Medicine, College of Veterinary Medicine, Gyeongsang National University, Jinju 52828, Republic of Korea; jaehcho@gnu.ac.kr (J.-H.C.); kimchi3237@gnu.ac.kr (C.-H.K.)

**Keywords:** dog, physical therapy, treadmill exercise, rehabilitation, TPLO

## Abstract

**Simple Summary:**

Most of the problems that appear after tibial plateau leveling osteotomy surgery are lameness. The dog exhibited mild lameness due to pain and inflammation, impeding full recovery. The purpose of this study is to collect objective data using walking analysis and digital thermal imaging to aid recovery from potential pain, inflammation, and lameness resulting from delayed rehabilitation after tibial plateau leveling osteotomy. The dog had not experienced rehabilitation until 42 h post-surgery. Gait analysis revealed insufficient weight support and the digital thermal imaging confirmed an increase in relative body heat within the medial stifle joint. After that, cold treatment, transcutaneous electrical nerve stimulation, and treadmill exercise were applied to promote recovery. Veterinary rehabilitation is often neglected post-surgery, despite its ability to enhance a dog’s quality of life.

**Abstract:**

A 7-year-old neutered Maltese dog weighing 5.1 kg was presented, with a tibial plateau-leveling osteotomy (TPLO) on the right hindlimb 42 days prior. The patient’s right hind limb showed lameness, intermittent limping, and atrophy, and the patient had not experienced rehabilitation since TPLO surgery. The patient showed a pain reaction at the end of the stifle extension, and an increased body temperature was identified on the medial side of the right hindlimb when compared with the left hindlimb using a digital thermal imaging device. In addition, a type of lameness, only partial weight bearing in the right hindlimb, was also identified during the gait analysis. The pain was relieved by applying a cold pack and transcutaneous electrical nerve stimulation, and the patient’s weak muscles were strengthened through treadmill exercises. In this study, physical therapy and rehabilitation exercises controlled pain and induced rapid recovery, indicating that rehabilitative intervention is required after TPLO surgery.

## 1. Introduction

Cranial cruciate ligament deficits progress to secondary osteoarthritis and are the most common orthopedic diseases to cause lameness and limping [1,2]. Tibial plateau leveling osteotomy (TPLO) is a surgical technique, designed in 1993, used to stabilize the stifle joint during weight-bearing, without repairing ligaments and with neutralization of the cranial drawer sign [3]. Similar to orthopedic surgery, intraoperative or postoperative complications involving soft tissues, bones, implants, or complex elements can occur with TPLO. The mean duration from surgery to the diagnosis of the first complication is 66.8 ± 10.8 days. Lameness and pain caused by joint capsule swelling, bruising in the surgical area, and seroma are minor complications that do not require additional surgical intervention [4,5]. After TPLO surgery, dogs that underwent 6 months of rehabilitation showed a faster recovery in reducing osteoarthritis progression, increasing activity levels, and improving the maximum vertical force (MVF) in the limb compared to dogs that did not undergo rehabilitation [6]. Digital thermal imaging devices have not only the advantages of providing quantitative imaging information of thermal alteration, but also of non-invasiveness, high versatility, and the absence of contamination. It has been used for tumor diagnosis and the evaluation of orthopedic and neurological disease [7]. When the body is inflamed, it is confirmed that the body temperature rises when compared with the contralateral side [8,9]. When a weight load occurs on the affected limb of a patient with lameness, the MVF on the affected limb decreases compared with that on the unaffected limb. After TPLO, subtle lameness is not visually apparent and may interfere with patient recovery. Most importantly, because the initial rehabilitation can fail with persistent lameness, rehabilitative intervention through gait analysis is essential for recovery.

## 2. Materials and Methods

### 2.1. Patient

A neutered 5.1 kg, 7-year-old Maltese dog, who underwent TPLO surgery 42 days prior for cranial cruciate ligament rupture of the right hind limb, presented for rehabilitation. After the surgery, the owner reported that the patient did not support weight well on the affected limb and seemed uncomfortable, had never undergone rehabilitation, and the owner was advised by a veterinary surgeon to perform passive range of motion exercises and walking at home for a short time. Based on video recordings of the dog walking and trotting, its lameness score was determined to be 1. The evaluation was performed using the method proposed by Millis and Levine [10]. There was no abnormality in the patient’s complete blood cell count at the time of surgery, and there were increases in alkaline phosphatase, alanine aminotransferase, and gamma-glutamyl peptidase and a decrease in creatinine in serum chemical analysis. Medical tests on the liver and biliary system were also conducted. Upon physical examination, there was second-grade medial patellar luxation on the left hindlimb. During the passive range of motion assessment, the patient exhibited discomfort and whined in pain at the end of the extension. Edema and discomfort were observed around the patellar tendon and medial patellar joint. The patient participated in rehabilitation treatment six times a month, and no internal medicine was prescribed.

### 2.2. Measurement

#### 2.2.1. Digital Thermal Imaging

The unaffected and affected sides in the front and medial views were simultaneously measured using a digital thermal imaging device (Digatherm^®^, IR-Pad 320, INFRARED CAMERAS INC., Beaumont, TX, USA) with a spectral band between 7 and 14 µm and a resolution of 336 × 256 pixels. The dog had limited exercise, was maintained in temperature-controlled runs, and was measured at room temperature and moisture (20–24 °C, 40–45%). All laboratory and radiographic evaluations, as well as physical examinations were conducted two days before thermal imaging to acclimate the dog and minimize the possible effects on his sympathetic nervous system that could alter thermal patterns. The dog was kept with his owner in a room designed to reduce stress and avoid physical responses for at least two hours. For better quality pictures, the owner held the front limbs while a detailed picture of the hind limb was taken at 50–60 cm from the camera to minimize environmental artifacts on the non-metallic platform.

#### 2.2.2. Gait Analysis

To establish a treadmill exercise plan, the evaluator asked the owner to leash the dog and walk as usual and the MVF and symmetry index (SI) were measured using a gait analysis device (FDM-TPROF CanidGait^®^, Zebris Medical GmbH, Allgäu, Germany) with dimensions of 212 × 60.5 cm (L × W) and a sensor surface of 203 × 54.2 cm. For each measurement, the dog walked three times for one minute, and the average was measured and generated on the screen for analysis. The velocity ranged from 4.3 to 5.3 km/h.

#### 2.2.3. Helsinki Chronic Pain Index

The HCPI evaluates chronic orthopedic pain in dogs based on 11 multifactorial descriptive scale questions that assess mood, behavior, and locomotion, as well as the emotional aspect of the pain [11,12]. It is used as an assessment tool for chronic pain at the beginning and end of rehabilitation. File S1 was cited in the Supplementary Materials.

#### 2.2.4. Physiotherapy and Rehabilitation

**Figure 1:**
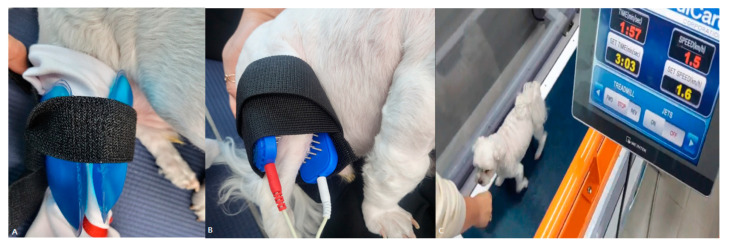
Physiotherapy and treadmill exercise. (**A**) After wrapping a thin towel around the right hindlimb, cold packs were applied to the medial and lateral sides of the stifle joint. (**B**) For TENS application, two electrodes were placed medial and lateral on the stifle joint, and the current was applied. (**C**) The speed was set at 1.5 km/h, and treats were used to encourage patient concentration and motivation.

In the first evaluation, using gait analysis, the difference in the MVF of the bilateral hind limbs was 34%. On the digital thermal imaging device, the medial body temperature of the stifle joint in the affected limb was 1.3 °C higher than that on the right side; thus, a cold pack was applied for 15 min before exercise (Figure 1A). Exercise in the form of comfortable walking on a treadmill was then performed for 5 min at a speed of 1.5 km/h followed by 3 min at rest. In total, four sets were applied (Figure 1B). After 5 min of rest, two electrodes were contacted medial and lateral on the stifle joint, using transcutaneous electrical nerve stimulation (TENS) (PT3010-P, S+B medVET GmbH, Babenhausen, Germany) to control pain, applied at 80–120 Hz and 25 µs for 20 min with high frequency and low intensity (Figure 1C). In dogs, to determine the threshold for electrical stimulation, the intensity should be carefully increased until the animal shows a reaction such as glancing at the electrodes or tensing the limb muscles, and then decreased slightly until the animal stops reacting.

A cold pack was applied for 15 min to reduce the temperature around the stifle joint.

During the second gait analysis, the difference in the MVF of the bilateral hind limbs was 26%; therefore, rehabilitation was administered as in the first treatment method. During the third gait analysis, it was confirmed that the difference in the MVF of the affected limb, compared with that of the unaffected limb, was slightly improved, to 18%, and physical examination showed a decrease in the pain reaction during full extension. A cold pack was applied for 20 min before the treadmill exercise, 5 min of treadmill exercise was conducted at 2.0 km/h, and then a 3 min rest was performed, in two sets. Then, 5 min of treadmill exercise was applied at 2.5 km/h, and a 3 min rest was performed, in two sets. TENS was applied in the previously described manner for 20 min, and a cold pack was applied again for 15 min.

During the fourth gait analysis, compared with the unaffected hindlimb, it was confirmed that the difference gradually improved to 9%. Treadmill exercise was conducted5 min at 2.0 km/h, and then a 3 min break was performed, in three sets. Then, 5 min of treadmill exercise was conducted at 2.5 km/h, and 3 min rest was performed. The TENS and cold packs were applied in the same manner as previously described.

During the fifth evaluation using a gait analysis device, it was confirmed that the affected limb showed a 4% difference, compared with the unaffected limb, and improved by 30% compared with evaluation before treatment. In the aforementioned manner, a cold pack was applied before the treadmill exercise, and the treadmill exercise started at 2.5 km/h and increased by 0.5 km/h three times, to 4.0 km/h, which was also applied for 5 min followed by rest for 3 min. There was no longer any pain response in the range of the stifle joint. Therefore, TENS was not applied, and only a cold pack was used.

During the sixth evaluation using the gait analysis device, it was confirmed that the MVF in the affected limb increased by 6% compared with the unaffected hind limb. The treadmill exercise proceeded with the same protocol as the fifth exercise, and the cold pack was also applied in the same manner. Rehabilitation was completed, and the owner was notified that it was the last treatment.

## 3. Results

### 3.1. Digital Thermal Imaging

**Figure 2:**
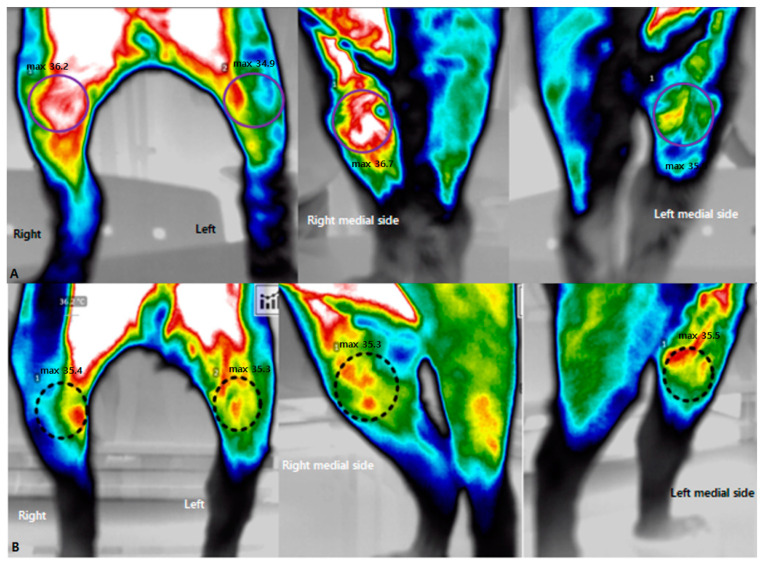
The application of digital thermal imaging before and after treatment. (**A**) Pre-treatment imaging of the affected side showed a higher body temperature than did the non-affected side on the anterior medial side around the stifle joint. (**B**) Post-treatment showed that there was no discernible difference in the body temperatures between the affected and non-affected sides in the medial view.

It was possible to evaluate changes in tissue perfusion for a specific area identified on a computer. The medial and lateral sides around the stifle joint were set as the regions of interest, and the affected and unaffected sides were compared. Before physiotherapy and rehabilitation, the patient’s body temperature around the stifle joint was measured using a digital thermal imaging device. As a result of measurement from the front, the temperature at the maximum point on the medial view of the right stifle was 36.2 °C, and that at the maximum point of the left stifle was 34.9 °C. The temperature at the medial side was also measured for a more detailed evaluation. A maximum temperature of 36.7 °C was measured on the medial side of the stifle joint of the right hindlimb, and a maximum temperature of 35 °C was measured on the medial side of the left stifle joint (Figure 2A). After the sixth physical therapy and treadmill exercises, the body temperature around the stifle joint in the front was measured at a maximum temperature of 35.4 °C on the right and 35.3 °C on the left. On the medial side, the maximum measured temperature of 35.3 °C on the right and 35.5 °C on the left. No significant thermal differences were observed (Figure 2B).

### 3.2. Gait Analysis

**Figure 3:**
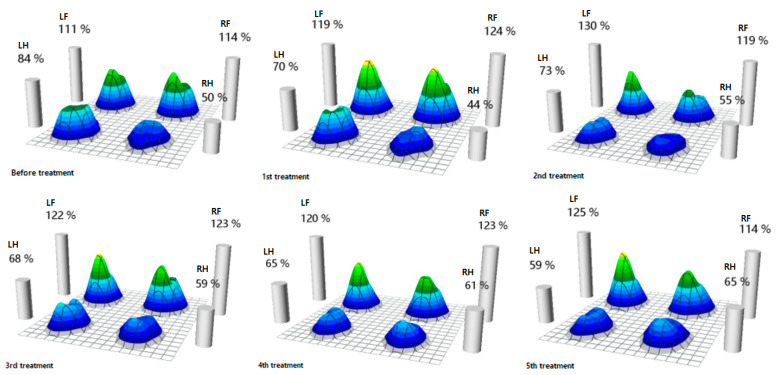
MVF of each limb from the beginning of rehabilitation to the end of rehabilitation. Percentage means of MVF are according to weight, and it was measured that the MVF in the affected hindlimb, compared to that of the non-affected hindlimb, gradually improves as the number of treatments increases (LF—left forelimb, RF—right forelimb, LH—left hindlimb, RH—right hindlimb).

Before physiotherapy and rehabilitation, the patient’s affected hind limb had a 34% decrease in MVF compared with the unaffected hind limb. As physical therapy and treadmill exercise were repeated, the MVF in both hind limbs improved in a balanced manner, so the difference in the MVF on the affected and unaffected hind limbs was only 6% during the fourth treatment. The MVF increased further in the right hind limb after the fifth treatment (Figure 3).

**Figure 4:**
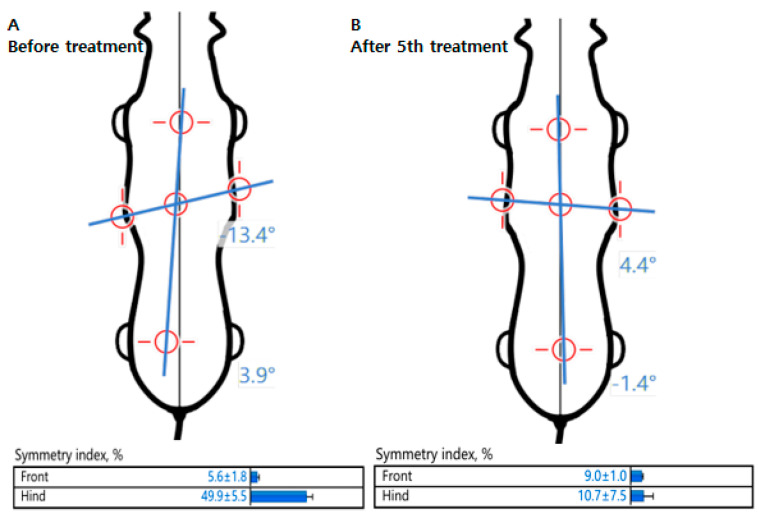
Evaluation of body symmetry and symmetry index. (**A**) The longitudinal axis is tilted to the left hindlimb before rehabilitation. (**B**) The longitudinal and transverse axes improved symmetrically after the 5th rehabilitation compared to pre-rehabilitation.

In addition, comparing pre- and post-physiotherapy and rehabilitation in terms of body symmetry, the longitudinal tilt was significantly reduced, shifting the axis to the center of the body. The SI also decreased after rehabilitation, confirming that the patient improved to a symmetrical gait (Figure 4).

### 3.3. The Helsinki Chronic Pain Index

Pain was also identified in the range of motion assessment, and the patient was evaluated as having serious pain by scoring 24 points on the HCPI before physiotherapy and rehabilitation. After physiotherapy and rehabilitation, the recorded HCPI score was 2.

All the results of the study are summarized in Table 1 below.

## 4. Discussion

Loss of function in the cranial cruciate ligament progresses in the order of gradual stretch, partial rupture, and complete rupture, and is the most common orthopedic problem in the hindlimbs of adult dogs [1,13]. The cruciate ligament serves as a primary stabilizer for the stifle joint to limit excessive forward displacement of the tibia to the femur by preventing internal rotation and hyperextension of the stifle joint [2,14]. 

In horses, the variable of body temperature change is caused by tissue vascularization and metabolism and showed symmetry; dogs show similar patterns. These results suggest that, when evaluating patients with unilateral diseases, the region of interest can be used as a control image for comparative purposes [15,16]. Based on previous research results, this study attempted to confirm the presence or absence of abnormalities using digital thermography imaging of the front and medial sides around the stifle joints of a dog with lameness after TPLO. As a result of measuring body temperature on the left and right sides in horses with asymmetrical swelling and lameness in the right hindlimb’s tarsal and metatarsal joints, temperature differences of 1.3 °C and 1.6 °C, respectively, compared with the contralateral side were measured, and cartilage damage was confirmed through an autopsy [9]. In humans with patellofemoral arthralgia, the anterior view of the knee shows an increase in body temperature on the medial side of the patella, whereas the medial view of the knee shows that this body temperature rise radiates from the patellar insertion of the vastus medialis into the muscle itself [17]. In this case, it was confirmed that the body temperature of the hindlimb affected with lameness was increased by 1.7 °C compared with the unaffected hindlimb, especially on the medial side. In TPLO surgery, this is considered to result from a temperature rise radiating to the surrounding tissues due to noninfectious inflammatory reactions caused by incomplete recovery of soft tissue and connective tissue. After the fifth rehabilitation, the maximum measured temperature was 35.4 °C on the right stifle joint and 35.3 °C on the left stifle joint in the front view, and the right and left stifle joint were measured at 35.3 °C and 35.5 °C, respectively, in the medial view. We confirmed that there was a slight difference. 

After the extracapsular technique, edema was most frequently reduced in the group for whom only cold compression was applied to reduce stifle joint edema [18]. Cold treatment is effective enough in reducing pain, edema, collagenolysis, synovial cystitis, and joint damage caused by chronic arthritic diseases to reduce the use of painkillers [10]. Cooling the tissue reduces inflammation, histamine secretion, tissue damage, and muscle spasms by lowering metabolic rates and firing rates of muscle spindles [19,20]. Unmyelinated cutaneous receptors for pain and temperature, known as Type IV or C receptors, exhibit sharp declines in firing frequency at temperatures below 20 °C and become minimal at 10–12 °C; thus, the effects of a 20 minutes cold pack treatment for dogs on skin and muscle temperatures, as well as intramuscular blood flow, were examined [10].

In this case, the increased body temperature identified on the affected hind limb through the digital thermal imaging device was regarded as inflammation of the tissue around the joint after TPLO, and this inflammation was alleviated by applying a cold pack, which helped reduce tissue damage.

Gait analysis can be used to evaluate the stabilization of the stifle joint before and after TPLO and is a useful method for objective and repetitive measurement of the limb. The Aβ fibers can be activated with a high-frequency electrical current (50 Hz or greater) at low current intensities, which is appropriate for pain control. Animals often do not tolerate high-intensity activities, and they are not recommended [10]. The SI for the MVF in the affected limbs of dogs subjected to intensive physical therapy, including diet for weight loss and TENS, was significantly improved over a period of 60–180 days of application [21]. In a dog with delayed rehabilitation for 6 months due to degenerative arthritis and sciatic nerve injury, symmetry improved after the seventeenth rehabilitation session [22]. When limbs are not used, cartilage, muscle, tendons, ligaments, and bone become weaker. To safely strengthen and remobilize these tissues, musculoskeletal rehabilitation must take this weakness into account [23]. In this case, cold packs and TENS were used for physical therapy, and the MVF and SI were confirmed using a gait analysis device. Conventional electrotherapy was applied, instead of low-frequency and high-intensity therapy, due to chronic pain, as the patient was sensitive and the owner refused sedation. Compared with the evaluation before treatment, it was confirmed that the MVF in the affected hindlimb was improved, and the SI was also improved symmetrically, as the difference in the MVF on the unaffected and affected hindlimb decreased. However, the increase in the MVF of the affected hind limb of the patient during the sixth gait analysis needs to be confirmed by repeated analyses. In addition, because the unaffected hind limb had second-grade medial patellar luxation, it can be considered that the MVF on the affected side was improved over that on the unaffected side. There was no significant difference in the two-point range between physical therapy and home exercise groups 6 weeks after TPLO surgery in terms of their lameness scores [24]. However, in this case, continuous lameness was observed for 6 weeks after surgery using a gait analysis device. The patient gradually resolved lameness through physical therapy and treadmill exercise.

The HCPI is a valid, reliable, and responsive tool for assessing the response to osteoarthritis treatment in dogs [11]. I total, 31.1% of dogs who scored more than 12 points in 253 HCPI questionnaires after cranial cruciate ligament surgery were continuously indicated for chronic pain with lameness [12]. Before performing physical therapy, the patient was suffering from chronic pain, with 24 points scored on the HCPI, and after six physical treatments and rehabilitation, the patient was confirmed to have improved significantly to 2 points, and the pain disappeared.

This study confirmed improvements using only thermal imaging and gait analysis. There is a limitation in that the improvement effect of the rehabilitative intervention was not evaluated using kinematics. It is thought that the effect of rehabilitation following TPLO can be clarified through various evaluation methods.

## 5. Conclusions

Rehabilitation is frequently overlooked in clinical veterinary care. It is important to closely monitor pain and lameness following cranial cruciate ligament surgery in order to prevent chronic problems and return the animal to daily activities quickly. It is hopeful that future advances in rehabilitation will make it possible for animals suffering from pain to widely benefit from rehabilitative intervention.

## Figures and Tables

**Table 1:** Results for a dog in affected hind limb before and after rehabilitation following tibial plateau leveling osteotomy.

	DTI (°C)	MVF (%)	SI (%)	HCPI (Points)
Before rehabilitation	36.2	50	49.9 ± 5.5	24
After rehabilitation	35.4	65	10.7 ± 7.5	2

DTI—digital thermal imaging, MVF—maximum vertical force, SI—symmetry index, HCPI—Helsinki chronic pain index.

## Data Availability

Not applicable.

## Supplementary Materials

The following supporting information can be downloaded at: https://www.mdpi.com/article/10.3390/ani13172778/s1, File S1: Helsinki Chronic Pain Index.
