# Supplementary material for: Effect of Rehabilitation in a Dog with Delayed Recovery following TPLO: A Case Report
Source: Animals (Basel). 2023 Aug 31;13(17):2778. doi: 10.3390/ani13172778 (PMC10486540; doi:10.3390/ani13172778)
Supplement: Supplementary file 1 [file animals-13-02778-s001.zip › Supplementary File S1.pdf]

Dog’s Name: Date:

# Helsinki Chronic Pain Index

Hielm-Bjorkman HK, Rita H, Tulamo R-M. Psychometric testing of the Helsinki chronic pain index by completion of a questionnaire in Finnish by owners of dogs with chronic signs of pain caused by osteoarthritis. Am J Vet Res. 70: 727 – 734, 2009.

(As translated from Finnish to English)

*Circle the pain and function description that best represents your dog’s behaviour:*

Rate your dog’s attitude and/or mood:

| 0 | 1 | 2 | 3 | 4 |
|---|---|---|---|---|
| Very alert | Alert | Neither alert nor disinterested | Disinterested | Very disinterested/ lethargic |

Rate your dog’s willingness to participate in play or interact:

| 0 | 1 | 2 | 3 | 4 |
|---|---|---|---|---|
| Very willing | Willing | Reluctant | Very reluctant | Does not participate or interact at all |

Rate your dog’s frequency in vocalization or discomfort behaviour (audible whining, grunting, yelping, or unusual licking):

| 0 | 1 | 2 | 3 | 4 |
|---|---|---|---|---|
| Never | Hardly ever | Sometimes | Often | Very often |

Rate your dog’s eagerness to walk:

| 0 | 1 | 2 | 3 | 4 |
|---|---|---|---|---|
| Very eager | Eager | Reluctant | Very reluctant | Does not want to walk at all |

Rate your dog’s ability and/or willingness to walk up and/or down stairs:

| 0 | 1 | 2 | 3 | 4 |
|---|---|---|---|---|
| Very willing/able | Willing/able | Reluctant | Very reluctant | Does not do stairs at all |

Dog's Name: Date:

## Helsinki Chronic Pain Index con't

Rate your dog's ability and/or willingness to run:

| 0 | 1 | 2 | 3 | 4 |
|---|---|---|---|---|
| Very willing/able | Willing/able | Reluctant | Very reluctant | Does not run at all |

Rate your dog's ability and/or willingness to jump (onto bed, couch, vehicle, etc):

| 0 | 1 | 2 | 3 | 4 |
|---|---|---|---|---|
| Very willing/able | Willing/able | Reluctant | Very reluctant | Does not jump at all |

Rate your dog's ease in lying down:

| 0 | 1 | 2 | 3 | 4 |
|---|---|---|---|---|
| Very easy | Easy | Neither easy nor difficult | Difficult | Very difficult |

Rate your dog's rising from a down position:

| 0 | 1 | 2 | 3 | 4 |
|---|---|---|---|---|
| Very easy | Easy | Neither easy nor difficult | Difficult | Very difficult |

Rate your dog's ease of movement after a long rest:

| 0 | 1 | 2 | 3 | 4 |
|---|---|---|---|---|
| Very easy | Easy | Neither easy nor difficult | Difficult | Very difficult |

Rate your dog's ease of movement during and/or after exercise/walks (tired, dragging feet, scuffing nails, lying down):

| 0 | 1 | 2 | 3 | 4 |
|---|---|---|---|---|
| Very easy | Easy | Neither easy nor difficult | Difficult | Very difficult |
